# Functional Reconstitution of a Tunable E3-Dependent Sumoylation Pathway in *Escherichia coli*

**DOI:** 10.1371/journal.pone.0038671

**Published:** 2012-06-12

**Authors:** Sean P. O’Brien, Matthew P. DeLisa

**Affiliations:** School of Chemical and Biomolecular Engineering, Cornell University, Ithaca, New York, United States of America; Center for Genomic Regulation, Spain

## Abstract

SUMO (small ubiquitin-related modifier) is a reversible post-translational protein modifier that alters the localization, activity, or stability of proteins to which it is attached. Many enzymes participate in regulated SUMO-conjugation and SUMO-deconjugation pathways. Hundreds of SUMO targets are currently known, with the majority being nuclear proteins. However, the dynamic and reversible nature of this modification and the large number of natively sumoylated proteins in eukaryotic proteomes makes molecular dissection of sumoylation in eukaryotic cells challenging. Here, we have reconstituted a complete mammalian SUMO-conjugation cascade in *Escherichia coli* cells that involves a functional SUMO E3 ligase, which effectively biases the sumoylation of both native and engineered substrate proteins. Our sumo-engineered *E. coli* cells have several advantages including efficient protein conjugation and physiologically relevant sumoylation patterns. Overall, this system provides a rapid and controllable platform for studying the enzymology of the entire sumoylation cascade directly in living cells.

## Introduction

Sumoylation is a eukaryotic post-translational modification that involves the covalent conjugation of the 11-kDa SUMO (small ubiquitin-related modifier) protein to a lysine residue in a target protein (for recent reviews of the sumoylation mechanism and its implications see [1], [2], [3], [4], [5], [6]). Cellular processes in which sumoylation is involved include cellular trafficking, channel and receptor regulation, regulation of transcription-factor activity, DNA repair and replication, chromosome dynamics, mRNA processing and metabolism, cellular replication, and cross-talk with ubiquitination. The mechanism of SUMO attachment resembles other ubiquitin-like conjugation pathways. Briefly, mature SUMO is first activated by a heterodimeric SUMO-activating enzyme, E1, before passing to the SUMO-conjugating enzyme, E2. Only one E2 appears to exist in most well studied organisms including human, yeast, rat, and mouse. Unlike with ubiquitination, sumoylation may proceed in an E3-independent manner. This notion is based on the observation that binding of the E2 Ubc9 to the consensus sequence Ψ-K-*X-*E (where Ψ is a hydrophobic residue and *X* is an arbitrary residue) present in a target protein is sufficient for sumoylation [7], [8], [9]. Furthermore, grafting of this consensus sequence to a protein not normally sumoylated will result in its sumoylation [8], [10], [11].

Given the apparent E3-independent nature of sumoylation, the existence of SUMO E3 ligases was initially challenged [12], although evidence hinted at their existence [6]. The involvement of E3 ligases in sumoylation has now been demonstrated [13], [14], [15]. However, while an E3 can enhance target sumoylation [10], [13], [15], [16], its role in substrate specificity and lysine selection remains debated. The crystal structure of SUMO-RanGAP1-Ubc9-Nup358 complex suggests the E3 merely aligns the E2-SUMO pair for optimal E2 binding and SUMO transfer without itself binding the target protein [17]. Interactions between the target protein and E3 appear to augment efficiency, but sumoylation depends solely upon E2 binding [17]. Furthermore, individual genetic knockout of the mammalian SUMO E3 ligases PIAS1 [18], PIASy [19], and PIASx [20] in mice does not affect global sumoylation patterns. Similarly in yeast, knockout of the E3 Siz2 does not affect global sumoylation, although the knockout of the E3 Siz1 attenuates robustness [13]. Further studies in yeast examining sumoylation of individual proteins confirm this trend in overlapping E3 function [10]. Differences in local concentrations rather than differences in target recognition may be the mechanism whereby E3 specificity is manifested *in vivo* but is absent *in vitro* [10].

Importantly, SUMO E3 ligases are not dispensable in the cellular context as the knockout of every E3 is lethal [10]. Furthermore, emerging evidence suggests that the E3 may play a role in target specificity. Several proteins are modified at nonconsensus sequences [4] and an E3 ligase, not an E2, may be responsible for this modification. For example, Siz1 is required for sumoylation of PCNA’s nonconensus K164 site [21]. Several studies have confirmed that the PINIT domain of the E3 is solely responsible for this K164 lysine specificity [10], [22]. Further, E3s tend to bias the particular SUMO isoform that is attached to the target protein [23].

Several groups have reconstituted E3-independent sumoylation cascades in *E. coli* [11], [24], [25]. These sumo-engineered *E. coli* systems have several advantages. First, endogenous levels of sumoylated protein in eukaryotic cells tend to be low [3]. Thus, purifying quantifiable amounts from these cells is difficult, whereas obtaining ample yields for study from *E. coli* is typically straightforward. Second, because *E. coli* lacks an endogenous sumoylation system, the pathway may be isolated up to the point of the E2 for study. However, these systems are not without shortcomings. E3-independent sumoylation itself occurs at quantifiable levels only for protein concentrations far exceeding physiological levels. While proteins are clearly sumoylated, the physiological relevance of the modified proteins is unclear. For example, Mencía and de Lorenzo observed attachment of poly-SUMO-1 chains to target proteins in *E. coli* [11]. Because SUMO-1 lacks the consensus sequence present on SUMO-2 and SUMO-3 [26], it is not believed to homo-polymerize. However, more recent *in vitro* studies have shown that SUMO-1 is capable of forming chains through non-consensus lysines [28], albeit to a far lesser extent compared to SUMO-2 and SUMO-3 [27]. The physiological relevance of such poly-SUMO-1 chains is unclear [29], and SUMO-1 itself may be more involved in chain termination of SUMO-2 and SUMO-3 rather than formation *in vivo* [30]. Along similar lines, the physiological significance of some sumoylation sites observed by Okada *et al.* using sumo-engineered *E. coli* is also unclear [25].

Here, we engineered an E3-dependent SUMO-conjugation system in *E. coli* that employs members of the mammalian PIAS E3 ligase family and, as a result, involves no observable poly-sumoylation of target proteins. Furthermore, because *E. coli* lacks organelles and an endogenous sumoylation pathway, our system provides an alternative *in vivo* context that is insulated from factors such as target localization, downstream interactions, and the diversity of sumoylated proteins that confound studies of E3s in eukaryotic cells. Finally, we show that addition of the E3 increases the efficiency of sumoylation, yielding as much as ∼5 mg/L of SUMO-modified proteins. This makes possible greater titers of specifically sumoylated target proteins for use in biochemical and structural characterization.

## Results

### A Tunable E3-dependent Sumoylation System

**Figure 1:**
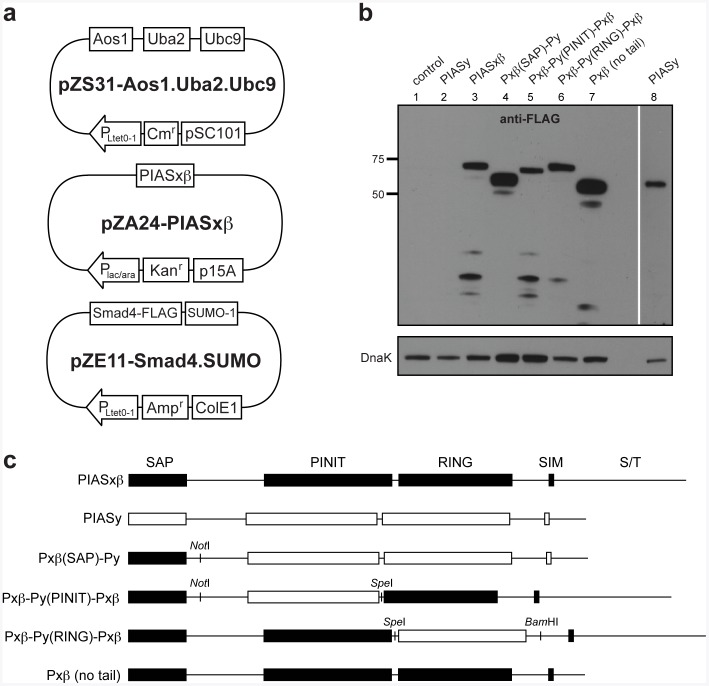
An E3-dependent sumoylation system. (a) Plasmid diagrams for the E3-dependent sumoylation system based on the pZ vector collection. The E1 (Aos1 and Uba2) and E2 (Ubc9) were cloned into the low copy plasmid pZS31-SMCS or the medium copy pZA31-SMCS (not shown); the E3 (e.g., PIASxβ) was cloned into the medium copy plasmid pZA24-SMCS; the target protein (e.g., Smad4-FLAG) and SUMO-1 were cloned into the high copy plasmid pZE11-SMCS. (b) Western blot analysis of cell lysate prepared from DH5α-Z1 cells expressing native and engineered SUMO E3 ligases as indicated. A much longer exposure time was required to visualize PIASy (lane 8). Control cells carried the empty pZE12-SMCS vector (lane 1). Blots were probed with anti-FLAG antibodies or anti-DnaK antibodies, with the latter serving as a loading control. (c) Schematic of the E3 chimeras and truncation mutant tested in this study. Chimeras were created by swapping different domains between human PIASxβ and PIASy using the inserted restriction sites.

To establish a SUMO-conjugation cascade in *E. coli*, the bacterial pZ vector system developed by Lutz and Bujard [31] was used. We chose the pZ vector system because of its modular nature, unique promoters, and medium to low copy number. Previous studies showed that strong expression of the E1 (human Aos1 and Uba2) and E2 (murine Ubc9) enzymes in *E. coli* results in sumoylation that is independent of the SUMO E3 ligase [11]. However, poly-sumoylated target evolves alongside mono-sumoylated product. To generate only mono-sumoylated target proteins, we attempted to reduce the expression of the E1 and E2 enzymes by placing the genes encoding human E1 and murine E2 into the medium copy vector pZA31-SMCS or the low copy vector pZS31-SMCS (Fig. 1a). To maximize sumoylated product, human SUMO-1 and the target protein were placed in the high copy vector pZE11-SMCS (Fig. 1a). A FLAG epitope tag was introduced to the C-terminus of the target protein to facilitate Western blot analysis. SUMO E3 ligases were placed on a separate plasmid, pZA24-SMCS, with a compatible replication of origin, p15A (Fig. 1a). The separate plasmid enables introduction of modifications to the E3 protein without altering the rest of the cascade components. Additionally, the P_lac/ara_ promoter allows modulation of the E3 expression level without impact upon the remaining components.

**Figure 2:**
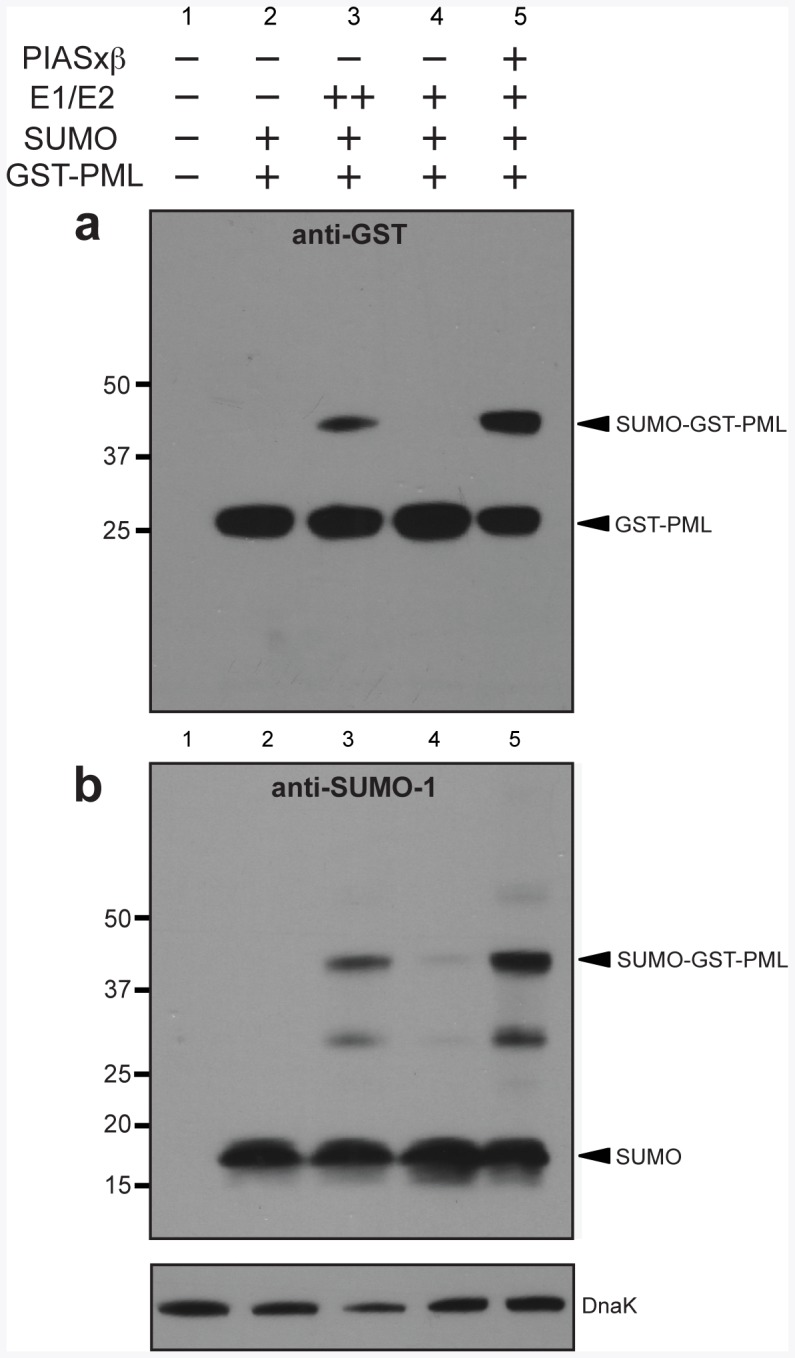
E3-dependent sumoylation of synthetic GST-PML. Western blot analysis of cell lysate prepared from DH5α-Z1 cells expressing the synthetic target GST-PML in the presence (+) or absence (-) of different SUMO-conjugation cascade components. The E1 and E2 enzymes were expressed from either the medium copy plasmid pZA31-SMCS (++) or the low-copy plasmid pZS31-SMCS (+). GST-PML was detected using anti-GST antibodies (a) while SUMO-1 was detected using anti-SUMO-1 antibodies (b). Detection of endogenous DnaK with anti-DnaK antibodies served as a loading control.

We first investigated the bacterial expression of several mammalian SUMO E3 ligases. Specifically, four enzymes from the PIAS family were tested (PIAS1, PIASxβ, PIAS3, and PIASy). Of these, PIASxβ was expressed most efficiently (Fig. 1b; data for PIASxβ and PIASy only); hence, we chose this E3 for further study. The synthetic GST-PML target of Mencía and de Lorenzo was chosen as a model target substrate for our E3-dependent SUMO-conjugation system [11]. This substrate is comprised of *E. coli* glutathione *S-*transferase (GST) that has been C-terminally modified with the 10-residue consensus sumoylation site from the promyelocytic leukemia (PML) protein. Previous studies using *E. coli* showed that this target can be sumoylated in an E3-independent manner [11]. In a similar fashion, we observed that when the E1 and E2 enzymes were expressed from the medium copy pZA31-SMCS vector in the absence of the E3, a slower migrating GST-PML band was detected (Fig. 2a, lane 3) but disappeared when the E1 and E2 were also absent (Fig. 2a, lane 2). Several lines of evidence indicate that this higher band is GST-PML that has become sumoylated in an E3-independent manner. First, this band migrated with an ∼20-kDa upshift compared to the unmodified GST-PML protein, which is consistent with the roughly ∼20-kDa shift previously reported for SUMO-1 [32]. Second, it reacted with anti-SUMO-1 antibodies (Fig. 2b, lane 3).

Next, we lowered the expression level of the E1 and E2 enzymes by inducing each from the low-copy pZS31-SMCS plasmid (Fig. S1). Under these conditions, the slower migrating band disappeared (Fig. 2a, lane 4). Given that a faint band was detectable upon probing with anti-SUMO-1 antibodies (Fig. 2b, lane 4), we conclude that sumoylation efficiency was drastically reduced under these conditions. Upon introduction of the SUMO E3 ligase PIASxβ? sumoylated GST-PML reappeared under conditions where the E1 and E2 were expressed from the low copy vector (Fig. 2a and b, lane 5 in each). Thus, by lowering the expression levels of the E1 and E2 enzymes and by adding a functional E3 enzyme, we successfully created an E3-dependent sumoylation cascade in *E. coli*. It is particularly noteworthy that the efficiency of sumoylation appeared to increase with the addition of PIASxβ (Fig. 2a or b, compare lanes 4 and 5). Although undetectable with anti-GST antibodies, a faint band above the mono-sumoylated GST-PML was observed using anti-SUMO-1 antibodies (Fig. 2b, lane 5). This band likely arises from GST itself becoming weakly sumoylated as has been previously reported [25]. The anti-SUMO-1 antibodies also revealed a ∼30-kDa band (Fig. 2b, lanes 3 and 5) that likely corresponds to a degradation product of the sumoylated target, a native *E. coli* protein that has become sumoylated, or a free di-SUMO-1 chain.

### Mono-sumoylation of Target Proteins by E3-dependent SUMO Modification System

**Figure 3:**
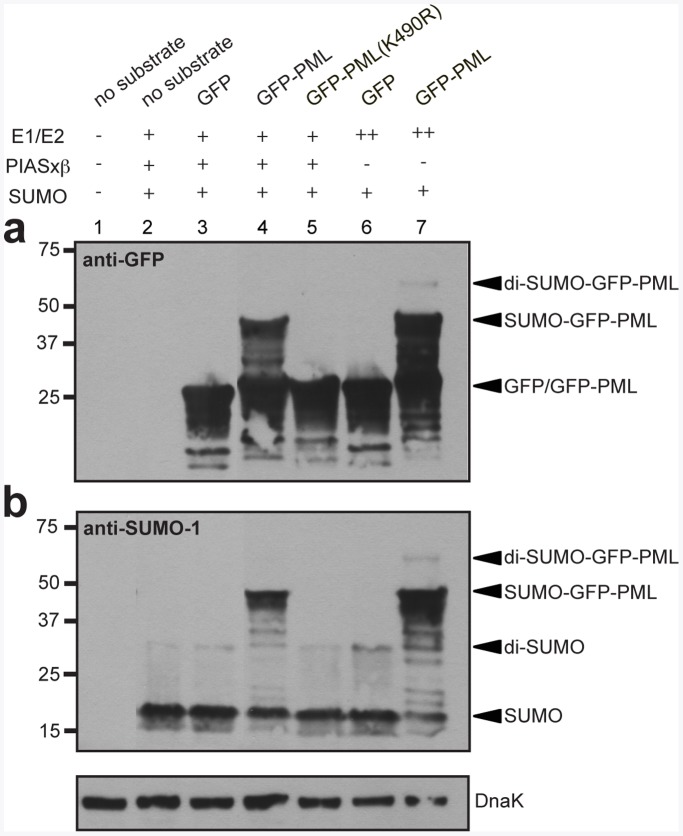
E3-dependent sumoylation of synthetic GFP-PML. Western blot analysis of cell lysate prepared from DH5α-Z1 cells expressing the synthetic target GFP-PML in the presence (+) or absence (-) of different SUMO-conjugation cascade components. The E1 and E2 enzymes were expressed from either the medium copy plasmid pZA31-SMCS (++) or the low-copy plasmid pZS31-SMCS (+). GFP was detected using anti-GFP antibodies (a), while SUMO-1 was detected using anti-SUMO-1 antibodies (b). K490 refers to the lysine’s native location within PML rather than its location within GFP-PML. Detection of endogenous DnaK with anti-DnaK antibodies served as a loading control.

In the earlier study of Mencía and de Lorenzo, E3-independent sumoylation in engineered *E. coli* resulted in modification of target proteins with SUMO-1 chains [11]. To more carefully investigate whether target proteins in our sumoylation system were poly-sumoylated, we converted the green fluorescent protein (GFP) to a sumoylation substrate by fusion to the PML tag. Since GFP does not contain any predicted sumoylation sites, mono- versus poly-sumoylation of the GFP-PML chimera can be used to assess SUMO-1 chain formation on target proteins. Indeed, expression of GFP without the PML tag in the E3-dependent (Fig. 3a and b, lane 3 in each) and E3-independent (Fig. 3a and b, lane 6 in each) systems resulted in no detectable target sumoylation. Likewise, no sumoylation was detected for the GFP-PML chimera when the lysine in the PML tag was mutated to arginine (Fig. 3a and b, lane 5 in each). On the other hand, expression of GFP-PML in the presence of the E3-dependent and E3-independent sumoylation cascades resulted in a clear band corresponding to mono-sumoylated GFP-PML. For the E3-dependent system, the yield of mono-sumoylated GFP-PML was ∼5 mg/L of culture (Fig. S2). Interestingly, a band corresponding in mass to di-SUMO-1 conjugated to GFP-PML was observed for the E3-independent but not the E3-dependent system (Fig. 3a and b, compare lanes 4 and 7 in each), suggesting that the addition of the E3 and/or the reduced expression of the E1 and E2 prevented poly-sumoylation. It is noteworthy that a rather faint ∼30 kDa band was detected with anti-SUMO-1 antibodies, similar to that seen above in the GST-PML experiments. Since this band did not depend on the presence of the target substrate (Fig. 3b, lanes 2, 3 and 6), we conclude that this band is not a degradation product of the sumoylated target. It is also noteworthy that the intensity of this band increased when the E1 and E2 were expressed from the medium copy plasmid and the E3 was absent (Fig. 3b, lanes 6 and 7).

### Conjugation of SUMO-1 to a Natural Sumoylation Target Protein

**Figure 4:**
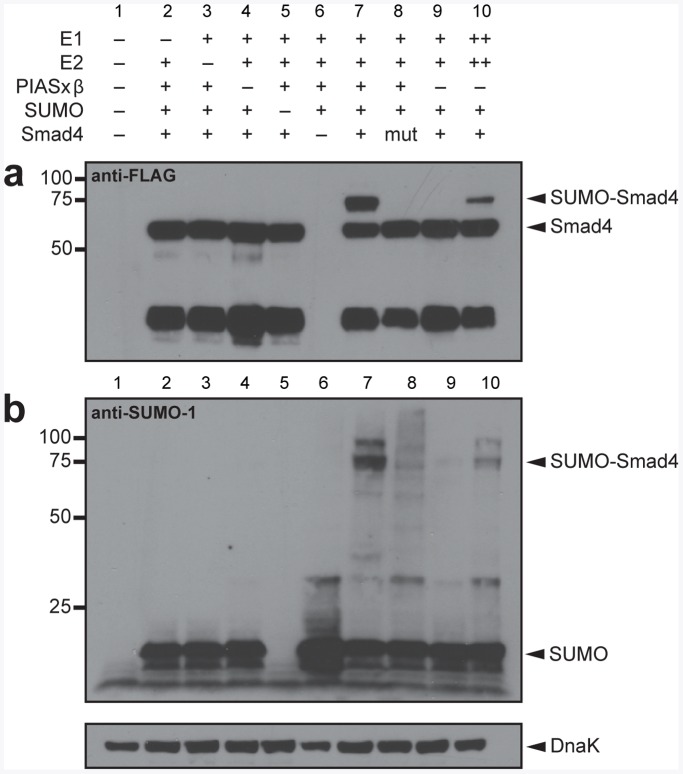
E3-dependent sumoylation of human Smad4. Western blot analysis of cell lysate prepared from DH5α-Z1 cells expressing human Smad4 or Smad4(K159R) (mut) in the presence (+) or absence (-) of different SUMO-conjugation cascade components. The E1 and E2 enzymes were expressed from either the medium copy plasmid pZA31-SMCS (++) or the low-copy plasmid pZS31-SMCS (+). Smad4 was detected using anti-FLAG antibodies (a), while SUMO-1 was detected using anti-SUMO-1 antibodies (b). Detection of endogenous DnaK with anti-DnaK antibodies served as a loading control.

Next, we investigated whether our sumo-engineered *E. coli* could conjugate SUMO-1 to a naturally occurring target of the human sumoylation machinery. We chose the human tumor suppressor protein Smad4, a central intracellular signal transducer for transforming growth factor-β (TGF-β) signaling, whose transcriptional potential is regulated by sumoylation [32], [33]. Similar to our results above, expression of the E1 and E2 from a medium copy vector resulted in E3-independent sumoylation of Smad4 (Fig. 4a and b, lane 10 in each) whereas expression of the E1 and E2 from a low-copy plasmid resulted in virtually no detectable Smad4 sumoylation (Fig. 4a and b, lane 9 in each). However, co-expression of the E1 and E2 from a low copy vector along with the E3 resulted in strong sumoylation of Smad4 (Fig. 4a and b, lane 7 compared to 1–6). As with the synthetic GST-PML, sumoylation of Smad4 appeared to be more efficient in the presence of the E3 (Fig. 4a and b, compare lanes 7 and 9). The major sumoylation site in Smad4 is the consensus lysine at position 159 [32], [34]. Mutation of this lysine residue to arginine (K159R) abolished Smad4 sumoylation (Fig. 4a, lane 8). To verify that K159 is the major site of SUMO attachment in our system, we performed MALDI-TOF mass spectrometry (MS) analysis on the SUMO-Smad4 band, which was purified on a Ni-NTA column and separated from unmodified Smad4 by SDS-PAGE. As expected, nearly all of the Smad4 was sumoylated at the consensus K159 (Fig. S3).

An even higher molecular weight band relative to SUMO-Smad4 was also produced in our sumo-engineered *E. coli* (Fig. 4a and b, lane 7 in each). This band might correspond to the attachment of SUMO-1 to a minor site on Smad4 or to the formation of SUMO-1 chains on Smad4. We favored the former possibility for two reasons. First, low-level expression of the E1 and E2 along with the E3 promoted mono-sumoylation in the case of GFP-PML. Consistent with this result, MS analysis of SUMO-Smad4 failed to reveal evidence for the formation of SUMO-1 chains at either K16 or K17 of the already conjugated SUMO-1 (data not shown). Second, a faint sumoylation band was observed for Smad4(K159R) (Fig. 4b, lane 8). Indeed, a known minor site of sumoylation on Smad4 is the non-consensus K113 residue [32], [34]. However, MS analysis did not provide any evidence for SUMO-1 conjugation at this position (data not shown). Thus, taken together, we suspect that another lysine is sumoylated on this higher molecular weight Smad4 species; however, at present the identity of this lysine remains undetermined.

### Functional Characterization of SUMO E3 Ligase Chimeras

**Figure 5:**
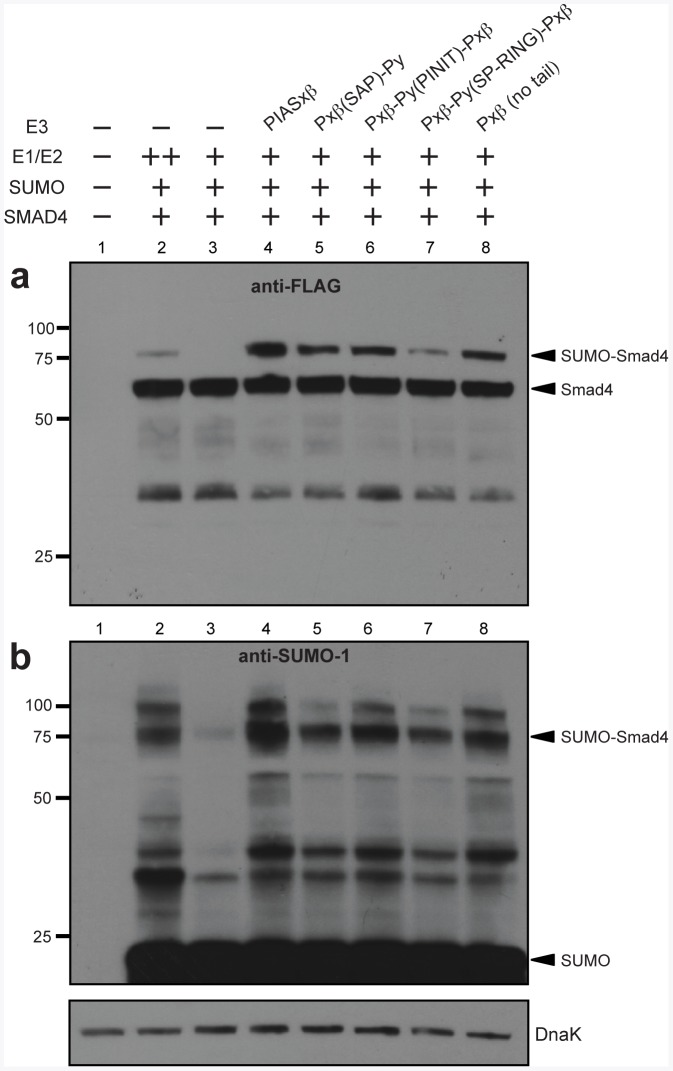
Chimeric E3-dependent sumoylation of human Smad4. Western blot analysis of cell lysate prepared from DH5α-Z1 cells expressing human Smad4 in the presence (+) or absence (-) of different SUMO-conjugation cascade components. The E1 and E2 enzymes were expressed from either the medium copy plasmid pZA31-SMCS (++) or the low-copy plasmid pZS31-SMCS (+). PIASxβ (lane 4) as well as a panel of E3 variants (lanes 5–8; see Fig. 1c caption for details) were tested for functionality. Smad4 was detected using anti-FLAG antibodies (a), while SUMO-1 was detected using anti-SUMO-1 antibodies (b). Detection of endogenous DnaK with anti-DnaK antibodies served as a loading control.

The generation of chimeras, truncations, and mutants of the Siz/PIAS protein family has provided great insight into the function of each protein [10], [35]. These alterations may impact localization, interaction with local cellular factors, and recognition of the target protein. However, decoupling these differences to deduce function can be difficult in the eukaryotic cellular environment. We hypothesized that our sumo-engineered *E. coli* could be useful for understanding the SUMO-conjugation activity of different E3 chimeras because it is devoid of the aforementioned complications. To test this notion, we constructed several SUMO E3 ligase variants. These included chimeras that were generated by swapping the SAP (scaffold attachment factor-A/B, acinus and PIAS), PINIT, or SP-RING (Siz/PIAS really interesting new gene) domains between PIASxβ and PIASy, and a truncation mutant that was made by eliminating the C-terminal tail of PIASxβ (Fig. 1c). Although PIASy expression could only be seen after a much longer exposure time compared to PIASxβ (Fig. 1b), its expression was second most efficient among all PIAS family members that were tested. Hence, we chose to use PIASy in our chimeric constructs. A panel of E3 variants which all contain the N-terminal SAP domain from PIASxβ were observed to express on par with PIASxβ (Fig. 1b). Since PIASy also sumoylates Smad4 [36], we predicted that each of these variants would sumoylate Smad4. In line with our hypothesis, all of the E3 variants conjugated SUMO to Smad4, albeit to varying extents (Fig. 5a and b, lanes 5–8 in each). None were as efficient as PIASxβ; the Pxβ-Py(RING)-Pxβchimeraappeared to be the least efficient (Fig. 5a and b, lanes 7 in each). Taken together, these data reveal the potential of our bacterial SUMO-conjugation system for functional evaluation of native as well as engineered SUMO E3 ligases.

## Discussion

In this study, we have created the first E3-dependent sumoylation system in *E. coli*. We anticipate that sumo-engineered *E. coli* will be useful in further studies of the sumoylation mechanism for several reasons. First, greater yields of sumoylated proteins for biochemical and structural analysis should be attainable through the addition of an E3 [13], [34]. Indeed, for both GST-PML and Smad4 substrates, we observed an increase in sumoylation efficiency following the addition of a functional E3 to the system. Furthermore, by lowering the expression of the E1 and E2, additional cellular resources can be diverted towards production of the target protein. Even without any process optimization, our E3-dependent SUMO conjugation system yielded ∼5 mg/L of mono-sumoylated protein. Second, the system enables functional characterization of any of the sumoylation cascade enzymes while eliminating the concern for localization, downstream interactions, and the diversity of sumoylated proteins that can obscure similar analysis in eukaryotic hosts. Our system also produces physiologically relevant results. For instance, we observed that Smad4 was sumoylated primarily at K159, which is reported to be the major sumoylation site [32], [34]. We did not detect sumoylation at position K113, which was reported as a minor site of sumoylation in one report [32] but was not sumoylated in another [37]. We also did not detect SUMO-1 chains on target proteins in our E3-dependent system, which is in stark contrast to an earlier bacterial E3-independent sumoylation system [11]. It should be noted, however, that the inability of MS analysis to reveal poly-sumoylation via K16 and K17 linkages on SUMO-1 could arise from low abundance and/or poor ionization efficiency of these species. Nonetheless, based on the high-intensity MS signal detected for the K159 SUMO-1 peptide, we conclude that no appreciable quantities of SUMO-1 chains are present. Overall, our system yields results that are entirely consistent with the known molecular biology of sumoylation. As a corollary, we show that engineered E3 variants can be expressed and functionally characterized in our system. This is significant because our bacterial SUMO-conjugation system provides a potentially less convoluted background for studying sumoylation. While *in vitro* reconstitution studies could also be used to eliminate these factors, our system obviates the need for purification of each cascade component and the corresponding need to modify each cascade component with a purification tag, which can affect enzyme function. Thus, we anticipate that our sumo-engineered *E. coli* system will be a useful new tool for illuminating the molecular details of the SUMO-conjugation process.

## Materials and Methods

### Plasmid Construction

All plasmids were based on the pZ vector system developed by Lutz and Bujard [31]. Primer insertions were used to replace the multiple cloning site (MCS) between the restriction sites *Eco*RI and *Xba*I in the plasmids pZE12, pZE11, pZA24, and pZS31. The resulting vectors - pZE12-SMCS, pZE11-SMCS, pZA24-SMCS, and pZS31-SMCS - consisted of three pairs of restriction sites (*Kpn*I and *Sph*I, *Mlu*I and *Eag*I, and *Kas*I and *Cla*I) with each pair flanked by a strong RBS sequence (5′ – AAAGAGGAGAAA –3′) and a frame-shifted stop codon sequence (5′ – TAATTGAATAGTTAA –3′) to prevent translational read-through. For any vector where these sites were not unique, we first cloned the genes into the modified pZE12 vector prior to moving the fragment generated by digestion with *Kpn*I and *Cla*I into the appropriate final vector. To make pZS31-Ubc9, pZS31-Aos1.Uba2, and pZS31-Aos1.Uba2.Ubc9, the genes encoding human Aos1, human Uba2, and murine Ubc9 were PCR amplified from pBADE12 [11]. The resulting PCR products were then inserted into pZS31-SMCS. For pZA31-Aos1.Uba2.Ubc9, pZS31-Aos1.Uba2.Ubc9 was cut at *Xho*I and *Cla*I and moved into pZA24-SMCS. The plasmid’s selection marker was changed to chloramphenicol using the restrictions sites *Spe*I and *Xho*I. A FLAG epitope tag for Western blot detection was introduced to Aos1 by adding the FLAG DNA sequence (5′ – GACTACAAGGACGATGACGACAAGGGA –3′) to the 3′ primer during PCR amplification. A 3×-FLAG epitope tag was added to Uba2 and Ubc9 using *Bsa*I and primer annealing of 5′– CTCAGACTACAAAGACCATGACGGTGATTATAAAGATCATGACATCGACTACAAGGATGACGATGACAAGTAAAT –3′ and 5′ –CGATTTACTTGTCATCGTCATCCTTGTAGTCGATGTCATGATCTTTATAATCACCGTCATGGTCTTTGTAGTC –3′. To generate the plasmids pZE11-GST-PML.SUMO, pZE11-Smad4-FLAG, pZE11-SUMO and pZE11-Smad4-FLAG.SUMO, GST-PML and human Smad4 were PCR amplified from pGST-PML [11] and pOTB7-Smad4 [40], respectively, and inserted between *Kpn*I and *Sph*I of pZE11-SMCS. DNA encoding a FLAG epitope tag was added C-terminally to Smad4 during PCR amplification. Human SUMO-1 was PCR amplified from pKRSUMO [11] and inserted between *Mlu*I and *Eag*I. The restriction site *Bsa*I was used to create the Smad4(K159R) mutant. To generate pZE11-GFP.SUMO, pZE11-GFP-PML.SUMO, and pZE11-GFP-PML(K490R).SUMO, GFP was PCR amplified and inserted between *Kpn*I and *Sph*I of pZE11-SUMO. For the latter two cases, DNA encoding PML or PML(K490R) was added C-terminally to GFP during PCR amplification. To construct pZA24-PIASxβ and pZA24-PIASy, PIASxβ and PIASy were PCR amplified from pCMV-FLAG-hPIASxβ [38] and pCMV-FLAG-hPIASy [39], respectively, and inserted between *Kpn*I and *Sph*I of pZA24-SMCS. To facilitate Western blot analysis, a FLAG epitope tag was added C-terminally to all of the E3s during PCR amplification.

To assemble the SUMO E3 ligase chimeras, fragments of PIASxβ and PIASy were PCR-amplified and restriction sites introduced during PCR amplification. The restriction sites were placed in predicted unstructured regions [41] that flanked a domain of interest and made use of silent mutations when possible to preserve the amino acid sequence. The restriction sites *Not*I, *Spe*I, *Bam*HI, and *Nhe*I were inserted after K79, L299, V436, and Q508 in PIASxβ, and after P77, L279, G440, and A509 in PIASy. Fragments containing these restriction sites were PCR amplified and then ligated together in plasmid pZE12-SMCS before being moved to pZA24-SMCS. PIASxβ was truncated after Q508 to create the PIASxβ truncation variant.

### Cell Growth and Western Blot Analysis

All constructs were transformed into *E. coli* host strain DH5α-Z1 [31] using a GenePulser Xcell (BioRad). Individual colonies were grown overnight in LB media with appropriate antibiotics (100 µg/mL ampicillin, 40 µg/mL kanamycin, and 12.5 µg/mL chloramphenicol) and then subcultured to OD_600_ ≈ 0.05 in 5 mL of fresh LB media supplemented with appropriate antibiotics. Cultures were induced at OD_600_ ≈ 0.75 with 0.5% L(+)-arabinose, 1 mM IPTG, and 50 ng/mL anhydrotetracycline when appropriate and subsequently shaken for 24 h at 16°C or 25°C depending on determined optimal conditions for sumoylation. Approximately 1.5 mL of each culture was harvested and lysed using 200 µL of Bugbuster Master Mix (Novagen) according to the manufacturer’s directions. Lysates were normalized to 10 µg of total protein as determined by a total protein assay (Bio-Rad) and loaded on a 4–20% Precise Protein Gel (Thermo Scientific). Transfers to Immobilon P Transer Membranes (Millipore) were performed for 2 h at the maximum amperage recommended for a Biosciences TE77 semi-dry transfer unit (Amersham). Blots were then imaged on film using standard protocols. The primary antibodies used were anti-GST (Abcam), anti-FLAG (Abcam), anti-GFP (Roche), anti-SUMO-1 (Abcam), and anti-DnaK (Stressgen). A standard curve was generated with purified GFP (AbCam) and used to quantify the yield of sumoylated GFP-PML. Densitometry analysis was performed on a Macintosh computer using the public domain NIH Image program (developed at the U.S. National Institutes of Health and available on the Internet at http://rsb.info.nih.gov/nih-image/).

### Protein Purification

Overnight cultures were subcultured into 250 mL of fresh LB media with appropriate antibiotics. At OD_600_ ≈ 0.5, cultures were induced as described above and shaken for 3 h at 37°C. Cells were then pelleted using a J2–21 floor centrifuge (Beckman) and lysed using Bugbuster Master Mix (Novagen). Samples were purified using Ni-NTA spin columns (Qiagen) according to the manufacturer’s instructions. Purification was not optimized.

### In-gel Digestion of Excised Gel Bands

Following visualization of the SDS-PAGE gel, two visible protein bands of interest were excised, diced, and placed into microtubes for the subsequent in-gel digestion and extraction. The in-gel digestion by chymotrypsin (from Sigma, St. Louis, MO) and the subsequent peptide extraction were performed following a protocol from Yang, *et al*. [42] with slight modification. The gel pieces were washed and destained with a series of solutions: 50 µL of water, 50 µL of 50% ACN/50% 50 mM ammonium bicarbonate pH 7.8, and 50 µL of 100% ACN. The samples were reduced with DTT and alkylated by treatment with iodoacetamide. Once the samples were dried down completely after washing, ∼0.2 µg LysC or chymotrypsin in 20 µL of 50 mM ammonium bicarbonate (pH =7.8) and 10% ACN was added to each tube. The samples were left on ice for 15 min and incubated overnight at 37°C. The supernatant containing digested peptides was removed after centrifuging for 2 min at 4000× *g*, and the remaining peptides were then extracted from the gel in a series of extraction steps. The first was with 30 µL of 25 mM ammonium bicarbonate pH 7.8 (30 minutes). Two sequential steps each with 50 µL of 5% formic acid in 50% acetonitrile (10 min) followed. For each extraction, the sample was sonicated for 5 min before the supernatant was removed. All gel-extracted supernatants were combined and evaporated to dryness in a Speedvac SC110 (Thermo Savant, Milford, MA).

### Protein Identification by nanoLC/MS/MS Analyses

The tryptic digest was reconstituted in 15 µL of 2% ACN with 0.5% FA for nanoLC-ESI-MS/MS analysis, which was carried out using a LTQ-Orbitrap Velos (Thermo-Fisher Scientific, San Jose, CA) mass spectrometer equipped with a nano ion source device (CorSolutions LLC, Ithaca, NY). The Orbitrap is interfaced with an UltiMate3000 MDLC system (Dionex, Sunnyvale, CA). The nanoLC was carried out by Dionex UltiMate3000 MDLC system (Dionex, Sunnyvale, CA). An aliquot of tryptic peptide (3.0 µL) was injected onto a PepMap C18 trap column (5 µm, 300 µm ×5 mm, Dionex) at a 20 µL/min flow rate for on-line desalting. It was then separated on a PepMap C-18 RP nanocolumn (3 µm, 75 µm×15 cm) and eluted in a 60 min gradient of 5% to 38% acetonitrile (ACN) in 0.1% formic acid at 300 nL/min followed by a 3-min ramping to 95% ACN-0.1%FA and a 5-min holding at 95% ACN-0.1%FA. The column was re-equilibrated with 2% ACN-0.1%FA for 20 min prior to the next run. The eluted peptides were detected by an Orbitrap through the nano ion source containing a 10-µm analyte emitter (NewObjective, Woburn, MA). The Orbitrap Velos was operated in positive ion mode with nanospray voltage set at 1.6 kV and source temperature at 225°C. Either internal calibration using the background ion signal at *m/z* 445.120025 as a lock mass or external calibration for FT mass analyzer was performed. The instrument was run at data-dependent acquisition (DDA) mode using FT mass analyzer for one survey MS scan followed by MS/MS scans on the five most intense peaks with multiple charged ions above a threshold ion count of 5000. MS survey scans were acquired at a resolution of 60,000 (fwhm at *m*/*z* 400) for the mass range of *m/z* 400–1400, and MS/MS scans were acquired at 7,500 resolution for the mass range of *m/z* 100 to 2000. Dynamic exclusion parameters were set at repeat count 1 with a 20 s repeat duration, exclusion list size of 500, 30 s exclusion duration, and ±10 ppm exclusion mass width. High energy dissociation (HCD) parameters were set at the following values: isolation width 2.0 *m/z*, normalized collision energy 45%, and activation time 0.1 ms. Xcalibur 2.1 operation software (Thermo-Fisher Scientific) was used to acquire all data.

### Data Analysis

All MS and MS/MS raw spectra were processed using Proteome Discoverer 1.1 (PD1.1, Thermo). The spectra from each DDA file were manually inspected for both expected precursor ions of interest and their MS/MS spectra. The mass accuracy for all identified peptides is within 2 ppm.

## Supporting Information

Figure S1 **Decreasing plasmid copy number reduces E1 and E2 expression levels.** (Top panels) Western blot analysis of cell lysate prepared from DH5α-Z1 cells expressing human Aos1 and Uba2 and murine Ubc9 from either the medium copy plasmid pZA31-SMCS (A) or the low-copy plasmid pZS31-SMCS (S). The protein name heading each column indicates the moiety bearing the epitope tag. Aos1 and Uba2 each bear a FLAG epitope tag while Ubc9 bears a 3×FLAG epitope tag as a single FLAG epitope was insufficient for detection. (Bottom panels) Same gel as in (a) but longer exposure time.
(PDF)

Figure S2 **Quantification of sumoylated GFP-PML by Western blot and densitometry analysis.** The amount of SUMO-GFP-PML produced by the entire sumoylation cascade (low copy expression of E1 and E2 plus the E3) was determined by direct comparison to known quantities of purified GFP standard as indicated. Densitometry analysis was performed on a Macintosh computer using the public domain NIH Image program (developed at the U.S. National Institutes of Health and available on the Internet at http://rsb.info.nih.gov/nih-image/).
(PDF)

Figure S3 (First panel) MS spectrum of Smad4 chymotryptic digests acquired in the FT analyzer of the Orbitrap Velos during the nanoLC-MS/MS analysis at elution time =23.71 min. A base-peak doubly-charged precursor ion at *m/z* 1109.9631 with its triply-charged ion at *m/z* 740.3111 shown in expanded view of insets is identified as sumoylated peptide. Sequence for the Smad4 peptide (red) with the conjugated SUMO-1 peptide (blue) after chymotrypsin digestion is shown. Lower case m indicates the oxidized methionine. The survey MS scan shows that the mass of the detected sumoylated peptide at K159 is under 1.8 ppm of its calculated mass. (Second panel) MS/MS spectrum of a triply-charged ion at *m/z* 740.31^3+^ acquired in HCD-DDA analysis by the FT analyzer at 23.90 min derived from Smad4 residues 149 to 162 with K159 identified as the sumoylated site. The y- and b-type ions are labeled in the spectrum as blue and red color for the SUMO-1 and the Smad4 target peptides, respectively. (Third panel) MS/MS spectrum of 1109.96^2+^ ion eluted at 23.84 min for identification of K159 sumoylation.
(PDF)
