# Supplementary figures and images for: Functional Reconstitution of a Tunable E3-Dependent Sumoylation Pathway in Escherichia coli
Source: PLoS One. 2012 Jun 12;7(6):e38671. doi: 10.1371/journal.pone.0038671 (PMC3373507; doi:10.1371/journal.pone.0038671)

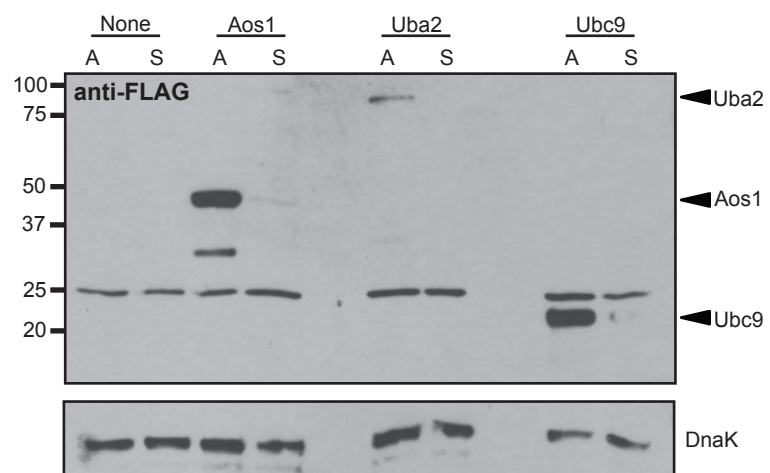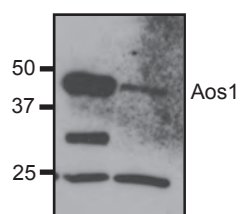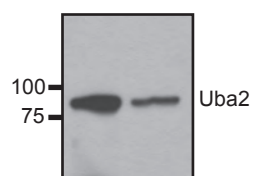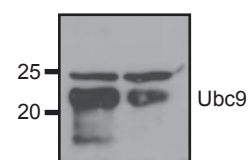

Supplement: Figure S1 — Decreasing plasmid copy number reduces E1 and E2 expression levels. (Top panels) Western blot analysis of cell lysate prepared from DH5α-Z1 cells expressing human Aos1 and Uba2 and murine Ubc9 from either the medium copy plasmid pZA31-SMCS (A) or the low-copy plasmid pZS31-SMCS (S). The protein name heading each column indicates the moiety bearing the epitope tag. Aos1 and Uba2 each bear a FLAG epitope tag while Ubc9 bears a 3×FLAG epitope tag as a single FLAG epitope was insufficient for detection. (Bottom panels) Same gel as in (a) but longer exposure time. (PDF) [file pone.0038671.s001.pdf]

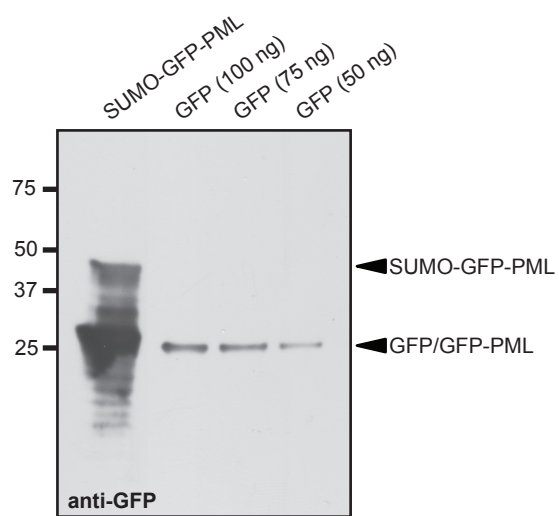

Supplement: Figure S2 — Quantification of sumoylated GFP-PML by Western blot and densitometry analysis. The amount of SUMO-GFP-PML produced by the entire sumoylation cascade (low copy expression of E1 and E2 plus the E3) was determined by direct comparison to known quantities of purified GFP standard as indicated. Densitometry analysis was performed on a Macintosh computer using the public domain NIH Image program (developed at the U.S. National Institutes of Health and available on the Internet at http://rsb.info.nih.gov/nih-image/). (PDF) [file pone.0038671.s002.pdf]

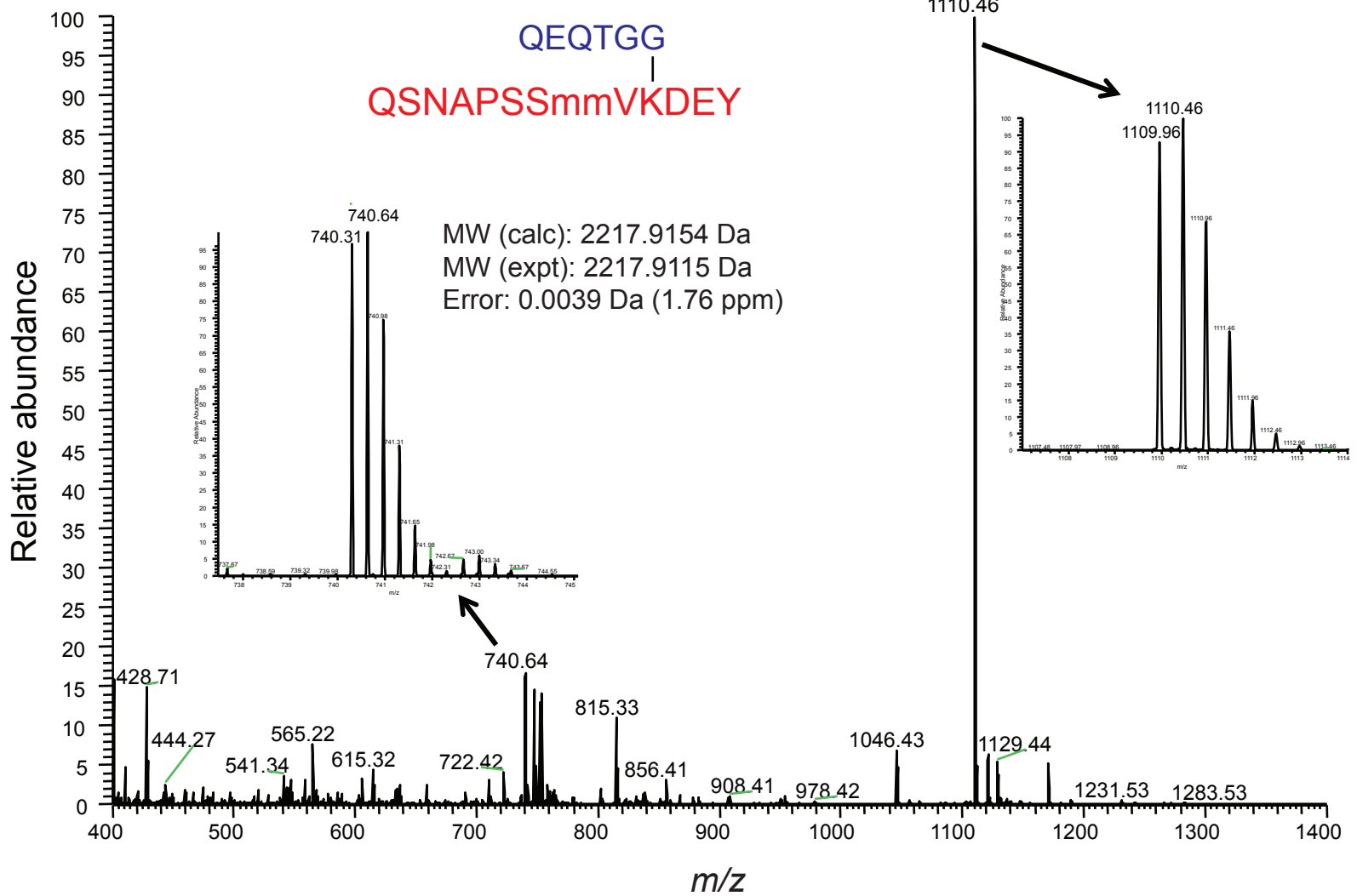

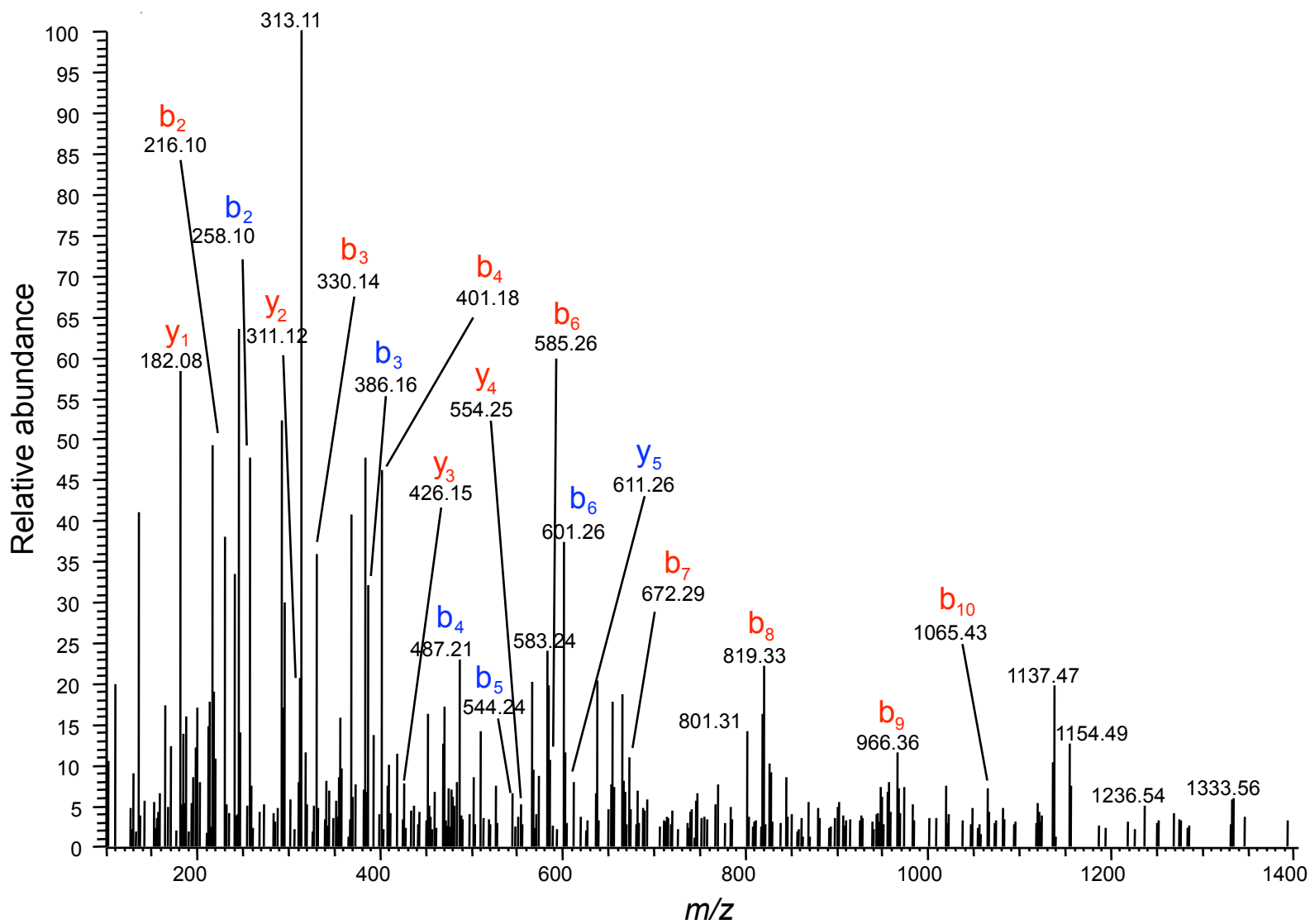

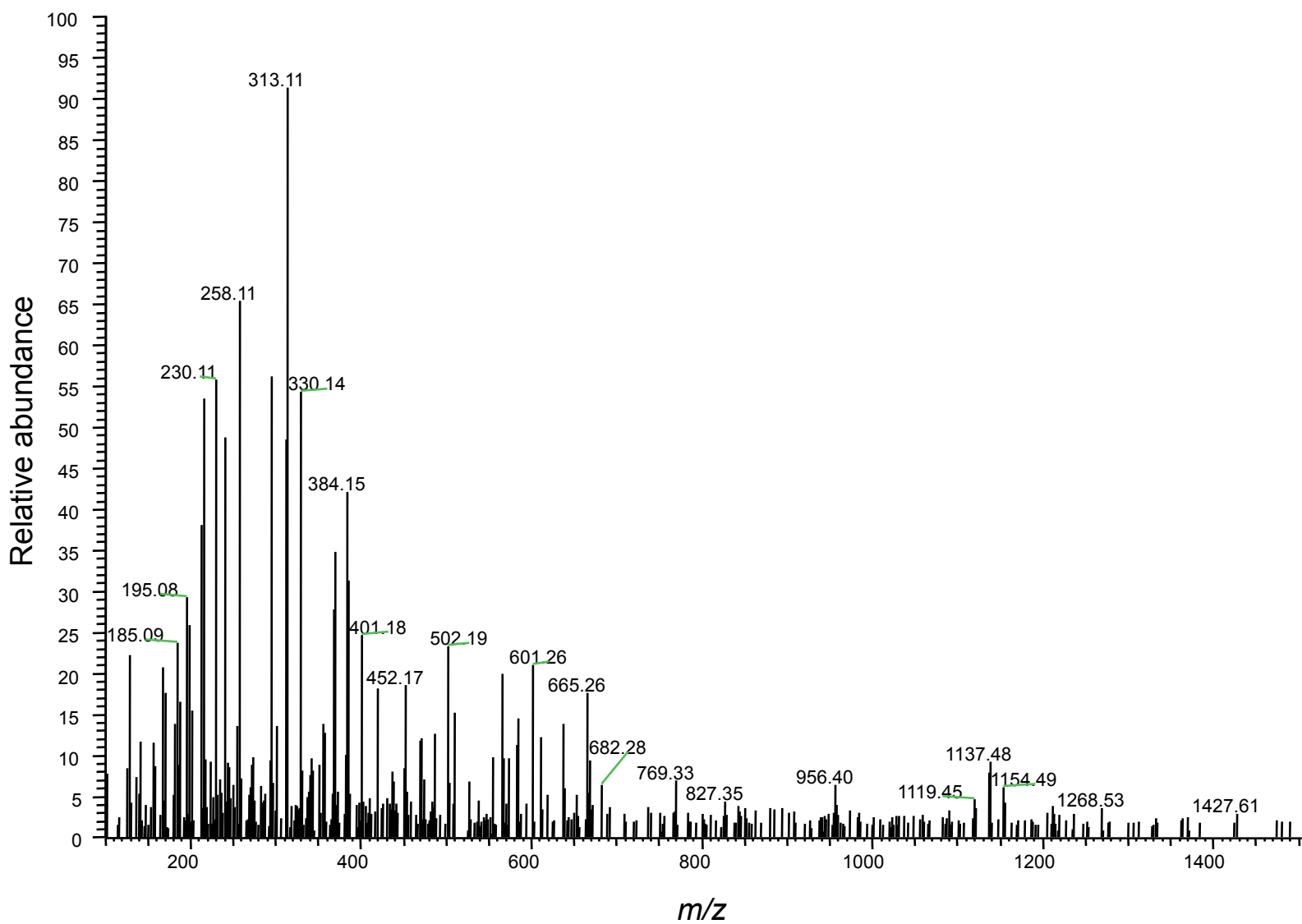

Supplement: Figure S3 — (First panel) MS spectrum of Smad4 chymotryptic digests acquired in the FT analyzer of the Orbitrap Velos during the nanoLC-MS/MS analysis at elution time =23.71 min. A base-peak doubly-charged precursor ion at m/z 1109.9631 with its triply-charged ion at m/z 740.3111 shown in expanded view of insets is identified as sumoylated peptide. Sequence for the Smad4 peptide (red) with the conjugated SUMO-1 peptide (blue) after chymotrypsin digestion is shown. Lower case m indicates the oxidized methionine. The survey MS scan shows that the mass of the detected sumoylated peptide at K159 is under 1.8 ppm of its calculated mass. (Second panel) MS/MS spectrum of a triply-charged ion at m/z 740.313+ acquired in HCD-DDA analysis by the FT analyzer at 23.90 min derived from Smad4 residues 149 to 162 with K159 identified as the sumoylated site. The y- and b-type ions are labeled in the spectrum as blue and red color for the SUMO-1 and the Smad4 target peptides, respectively. (Third panel) MS/MS spectrum of 1109.962+ ion eluted at 23.84 min for identification of K159 sumoylation. (PDF) [file pone.0038671.s003.pdf]
